# Mini-Laparoscopic Cholecystectomy During the Late Second and Third Trimesters of Pregnancy

**DOI:** 10.7759/cureus.63804

**Published:** 2024-07-04

**Authors:** Hadrien Tranchart, Celeste Del Basso, Alexandre Vivanti, Martin Gaillard, Alexandra Benachi, Ibrahim Dagher

**Affiliations:** 1 Minimally Invasive Digestive Surgery, Antoine-Béclère Hospital, Clamart, FRA; 2 School of Medicine, Paris-Saclay University, Orsay, FRA; 3 Obstetrics and Gynecology, Antoine-Béclère Hospital, Clamart, FRA

**Keywords:** minimally invasive laparoscopy, pregnancy, laparoscopy, mini-laparoscopy, cholecystectomy

## Abstract

Background: Mini-laparoscopic cholecystectomy (MLC) reduces abdominal wall injury and has the advantage of not altering the surgical principles of conventional laparoscopic cholecystectomy. Our team recently decided to extend the indications for mini-laparoscopy to pregnant women requiring cholecystectomy in the late second and third trimesters when significant uterine height is a potential difficulty.

Methods: From January 2022, all patients who underwent MLC after five months of pregnancy were included in the analysis. Operative, postoperative, and perinatal outcomes were retrospectively collected.

Results: Ten patients underwent MLC between 24 and 32 weeks of gestation. The mean operative time was 73 ± 24 minutes. Only one minor intraoperative complication was observed. The mean postoperative pain, quality of life, and cosmetic satisfaction at the first postoperative visit were 1.8 ± 0.9, 9.1 ± 0.8, and 9.5 ± 0.8, respectively. Finally, all patients had an uncomplicated vaginal delivery. No preterm delivery or fetal loss occurred.

Conclusions: These preliminary results suggest that the mini-laparoscopy could be used safely in selected pregnant women requiring cholecystectomy even after five months of gestation. By preserving the abdominal wall as much as possible, MLC may be of particular interest in this particular case, when the patient's abdominal wall could potentially be subjected to severe secondary stress in the event of vaginal delivery.

## Introduction

Gallbladder disease is highly prevalent in Western countries. Female sex and pregnancy are associated with a higher risk, and the incidence of biliary sludge and gallbladder stones during pregnancy was found to be 12% [1]. Up to 3% of pregnant women in America require a cholecystectomy in the first year after delivery [1]. Pregnancy itself is a pro-lithogenic state in which estrogen level increases biliary cholesterol production and progesterone reduces bile acid secretion and impairs gallbladder emptying [2]. Surgery treatment during pregnancy has historically been discouraged, especially during the first or third trimesters, due to a perceived high risk of fetal loss [3]. However, non-surgical strategies have been associated with higher rates of symptom recurrence and repeat hospitalizations. In addition, medically managed women have a higher rate of labor induction, preterm delivery, cesarean delivery, and relapse before delivery [4]. Finally, non-operative management of symptomatic cholelithiasis can increase the risk of gallstone pancreatitis, which can cause fetal loss in 10-20% of cases [5]. Since 2007, the Society of American Gastrointestinal and Endoscopic Surgeons (SAGES) has recommended early surgical treatment for pregnant women with symptomatic gallbladder disease regardless of trimester [6]. However, the second trimester is generally considered the most suitable period to perform laparoscopic cholecystectomy [7]. After that, laparotomy may be necessary in the case of difficult exposure, and several authors still advocate postponing surgery to the postpartum period if possible [8].

To enhance the efficacy of conventional four-port laparoscopic cholecystectomy (CLC), surgeons have endeavored to mitigate parietal injury by reducing the number and/or size of incisions. Various mini-invasive techniques for cholecystectomy have been documented, including three-port [9] or two-port [10] cholecystectomy, single-port cholecystectomy [11], or natural orifice endoscopic surgery (NOTES). Mini-laparoscopic cholecystectomy (MLC) offers several potential advantages over conventional laparoscopic cholecystectomy including improved aesthetic results, reduced risk of incisional hernia, and potential reduction in postoperative pain, which may have an impact on the postoperative period, such as in terms of analgesic consumption. It does not modify the surgical principles of conventional laparoscopic cholecystectomy, allowing for standardization and a short learning curve. In addition, mini-laparoscopic instruments are reusable and can be used for other types of laparoscopic surgery.

Since 2009, the MLC technique has been our standard approach for the majority of patients undergoing elective cholecystectomy [12]. More recently, we have decided to use this approach in women with symptomatic gallstones at the end of the second and third trimesters of pregnancy, when significant uterine height is a potential difficulty in performing cholecystectomy via a minimally invasive approach. The main idea was to minimize laparoscopic trauma to the abdominal wall in these patients. Indeed, during pregnancy, the abdominal wall is already weakened by structural adaptations, and we felt that it was important to preserve the abdominal wall during the cholecystectomy as much as possible, given that these patients would soon be subjected to significant physical stress during childbirth. In addition, we believed that the use of thin instruments could even help to perform surgical procedures in limited areas of the abdominal cavity. The aim of this retrospective study was to report our experience with MLC during the late second and third trimesters of pregnancy.

## Materials and methods

From January 2022, we started to use MLC in Antoine-Béclère Hospital (Clamart, France) in pregnant women with symptomatic gallstone disease (biliary colic, patients with passed common bile duct (CBD) stones, or cooled-off acute cholecystitis). All patients were informed about perioperative and intraoperative management, and informed consent was obtained before surgery. Patients with acute cholecystitis or chronic cholecystitis, recent necrotic pancreatitis, or requiring concomitant surgical extraction of CBD stones were not proposed for MLC. Preoperative biliary magnetic resonance imaging (MRI) is performed when there is doubt about the presence of a CBD stone (recent biliary pancreatitis, abnormal liver function tests, or ultrasound visualization of biliary dilatation) to avoid the need for intraoperative cholangiography, as usually performed in our center. Procedures were carried out in accordance with current French guidelines for surgery and anesthesiology in pregnancy. The use of tocolytics pre- or postoperative was decided on a case-by-case basis. Fetal heart rate was checked pre- and postoperatively from viability. Patients operated on before the sixth month of pregnancy were not included in the analysis.

Surgical technique

Induction of general anesthesia was started only when the surgical team was present in the operating room, in order to minimize operating time. Rapid-sequence induction using propofol or thiopental was used to minimize the risk of aspiration. Intraoperative pain management was provided with acetaminophen and sufentanyl. No steroidal or non-steroidal anti-inflammatory drugs were used. The mean arterial pressure was maintained above 90% of preoperative pressure, using noradrenaline if necessary. Intraoperative fetal monitoring was not used.

**Figure 1:**
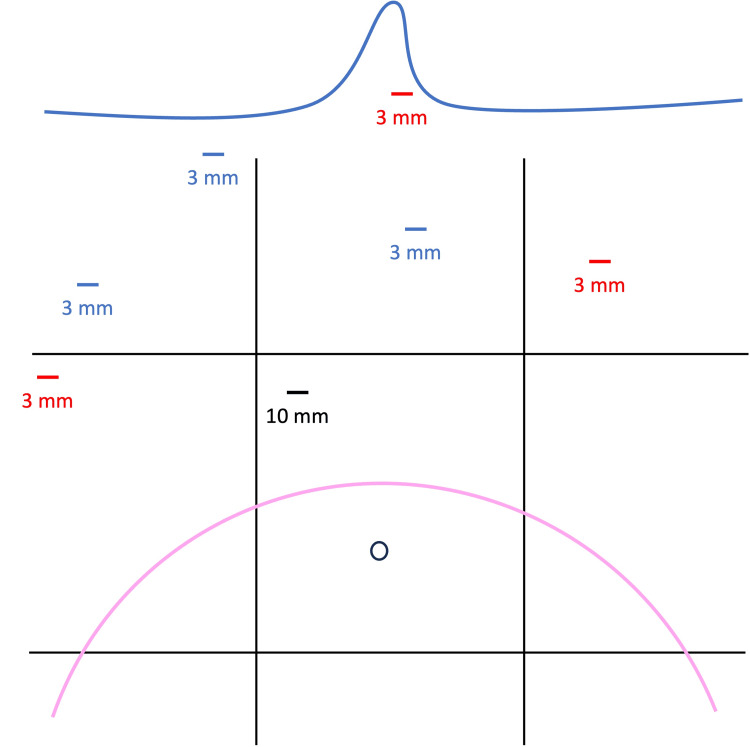
Schematic representation of trocar placement in the 9 abdominal regions during mini-laparoscopic cholecystectomy in women after five months of pregnancy. The 10 mm optical trocar (black) is placed using an open technique at the junction of the umbilical and right hypochondrial regions. The position of this trocar is adjusted according to the fundal height (pink) but is generally about 10 cm above the level of the umbilicus. Position of mini-laparoscopic trocars when using 30 cm (red) or 20 cm (blue) instruments.

Patients were placed in the supine position, legs apart with a partial left lateral decubitus position to minimize caval compression. The principal operator stands between the patient’s legs. Safe abdominal access is especially important in these patients. Therefore, an open technique is used to place a 10 mm trocar in the right hypochondrium. This trocar placement is of course adjusted according to the fundal height but generally performed approximately 10 cm above the umbilical level. A pneumoperitoneum of a maximum of 12 mmHg was progressively insufflated to allow an adequate visualization without over-compressing the already limited lung volume. When available, a continuous pressure insufflator of warmed and humidified CO_2_ allowing to work with a low pressure (9-10 mmHg) but stable pneumoperitoneum was used (AP 50/30®, Lexion Medical, St Paul, MN, USA). Three 3 mm manipulation trocars are used. Mini-laparoscopic instruments are available in standard length (30 cm) or short length (20 cm) (AB Medica, Méry-sur-Cher, France). The shorter instruments have the advantage of being less flexible but require the trocars to be positioned closer to the gallbladder (Figure 1).

Furthermore, when shorter instruments are employed, the epigastric trocar is positioned to the right of the round ligament in order to permit the gallbladder fundus to be displaced toward the right upper quadrant without tensioning the round ligament. Calot’s triangle is dissected with a cautery hook. The cystic duct and artery are controlled with 10 mm absorbable clips (Laproclip®, Medtronic, Paris, France), inserted through the 10 mm trocar, and positioned under visual control via a 3 mm optical device. Thus, two optical devices (10 mm and 3 mm) are alternatively used using a sterile camera head adaptor. The gallbladder is dissected from the liver bed in a retrograde fashion with the hook. In the event that additional instrumentation is required, a 3 mm bipolar coagulator and suction instruments may be employed. At the conclusion of the procedure, 20 mL of ropivacaine 0.5% (5 mg/mL) is administered around the trocar sites. The 3 mm skin incisions may be closed with staples, biological glue, or a simple adhesive. A brief video demonstrating the MLC technique in the late second trimester of pregnancy in a woman with obesity is available (Video 1).

**Video 1:** Mini-laparoscopic cholecystectomy in the late second trimester of pregnancy in an obese patient.

Postoperative follow-up

The patients were systematically hospitalized postoperatively in a high-risk pregnancy monitoring unit. Daily clinical monitoring was provided by a midwife and an obstetrician. Ultrasound was not routinely performed after surgery. After authorization by the obstetrical and surgical teams, the patients were discharged and returned to their usual care, which was systematically provided by an obstetrician. Postoperative pain was treated with group I or II oral analgesics, which are continued, if necessary, for one week after surgery. The patients were seen by the surgeon 10-15 days after the operation, three months after delivery, and one year after the operation. 

Statistical analysis

All operative and perioperative data for patients were retrospectively collected. The surgical parameters evaluated included operative duration, conversion to conventional laparoscopy rate, and intraoperative morbidity (including gallbladder perforation or bleeding, defined as bleeding from the hepatic pedicle or gallbladder bed requiring the use of aspiration/lavage). Postoperative data evaluated included early postoperative surgical morbidity (i.e., within 90 days after the operation), duration of hospital stay, and postoperative pain at discharge and/or at the first postoperative visit (assessed by a visual analog scale ranging from 0 to 10). Quality of life and cosmetic satisfaction were also measured with a visual analog scale (ranging from 0 to 10) at the first postoperative visit. Finally, perinatal outcomes including preterm delivery, mode of delivery, and maternal and fetal morbidity were evaluated.

Statistical analysis was performed using the IBM SPSS Statistics software (IBM Corp., Armonk, NY). Qualitative data were expressed as mean ± standard deviation (SD). Quantitative data were expressed as percentages.

## Results

Ten patients with singleton pregnancy underwent MLC between 24 weeks and 32 weeks of gestation. No patient underwent surgery after 32 weeks of gestation, as the obstetrical team opted for delivery first at this stage. The mean age of the patients was 26 ± 4 years old and the mean body mass index was 29.7 ± 5.2 kg/m^2^. No patient had a history of upper abdominal surgery, and all had an American Society of Anesthesiologists score of 2. The operative indication was biliary colic in eight patients and stone migration in two patients. Concerning patients with stone migration, one had a biliary MRI that showed choledocolithiasis and was addressed before surgery to endoscopic ultrasound that demonstrated a recent passage of stones through the ampulla of Vater without residual common bile duct stone. The other one had a preoperative negative biliary MRI.

The preoperative uterine height was 27.2 ± 3.2 cm. The mean operative time was 73 ± 24 minutes. No change in the number or size of trocars was necessary, and no patients required conversion to open surgery. Only one patient had an intraoperative complication, a gallbladder perforation with limited bile contamination, which had no postoperative consequences. No bleeding was noted. Postoperative surgical morbidity was nil. The mean length of hospital stay was 3.9 ± 0.8 days. The mean postoperative pain at discharge and at the first postoperative visit was 2.8 ± 0.9 and 1.8 ± 0.9, respectively. The mean quality of life and cosmetic satisfaction at the first postoperative visit were 9.1 ± 0.8 and 9.5 ± 0.8, respectively. 

All patients had uncomplicated vaginal deliveries. No preterm delivery or fetal loss occurred, and globally the maternal and fetal morbidity was nil. The mean birth weight was 3.4 ± 0.5 kg, and all children had a five-minute Apgar score of 10. Operative and perioperative data are summarized in Table 1.

**Table 1:** Operative and perioperative data of the 10 patients who underwent mini-laparoscopic cholecystectomy during the late second and third trimesters of pregnancy. ASA: American Society of Anesthesiologists

Characteristics	Mini-laparoscopic cholecystectomy (n = 10)
Age (years), mean ± SD	26 ± 4
Body mass index (kg/m^2^)	29.7 ± 5.2
Previous history of upper abdominal surgery, n (%)	0
ASA score = 2, n (%)	10 (100)
Surgical indication, n (%)	
Biliary colic	8 (80)
Stone migration	2 (20)
Preoperative uterine height (cm), mean ± SD	27.2 ± 3.2
Operative time (minutes), mean ± SD	73 ± 24
Additional trocar, n (%)	0
Trocar size modification, n (%)	0
Intraoperative complication, n (%)	1 (10)
Postoperative complication, n (%)	0
Length of stay (days), mean ± SD	3.9 ± 0.8
Postoperative pain	
At discharge, mean ± SD	2.8 ± 0.9
At the first postoperative visit, mean ± SD	1.8 ± 0.9
Quality of life at the first postoperative visit, mean ± SD	9.1 ± 0.8
Cosmetic satisfaction at the first postoperative visit, mean ± SD	9.5 ± 0.8
Term of delivery (weeks), mean ± SD	38 ± 1
Vaginal delivery, n (%)	10 (100)
Fetal or maternal morbidity, n (%)	0
Birth weight (kg), mean ± SD	3.4 ± 0.5
Five-minute Apgar score = 10, n (%)	10 (100)

## Discussion

Our results suggest that mini-laparoscopy could be used safely in selected pregnant women, especially at the end of the second and third trimesters. The use of thin laparoscopic instruments did not complicate the operation and may even be of interest to work in a reduced space in the abdominal cavity. Moreover, by preserving the abdominal wall as much as possible, mini-laparoscopy may have an impact, albeit limited, on simplifying obstetrical outcomes.

The issue of reducing postoperative pain, and therefore analgesic consumption, is fundamental even in minor abdominal surgery when a worrying rate of opioid addiction has been reported after cholecystectomy [13]. Mini-laparoscopy is part of a multimodal management approach that can help improve the already very good results of conventional laparoscopy in terms of pain management with systematic low-pressure pneumoperitoneum work or trocar port infiltration. The range of instruments available for mini-laparoscopy has improved considerably in recent years, particularly in terms of robustness, allowing the use of thin instruments (3.5 mm), even of standard size (30 cm), with efficacy fully comparable to that of conventional laparoscopic instruments. Mini-laparoscopic instruments have been successfully used for various types of abdominal surgery in adults, especially in gastrointestinal surgery, mainly for cholecystectomy, but also for appendectomy [14] and inguinal hernia repair [15]. In a meta-analysis, Coletta et al. showed that MLC did not increase postoperative morbidity and observed a significant reduction in postoperative pain in a pooled analysis that was not, however, confirmed in studies looking at the first postoperative day. This trend toward pain reduction was achieved at the cost of a very small increase in operative time (five minutes on average) [16].

Mini-laparoscopy, used as part of a multimodal approach to reduce postoperative pain, has been shown to reduce postoperative morbidity in the specific case of sickle cell patients, who are known to be fragile [17]. Our results suggest that the use of mini-laparoscopy could also be of interest in the particular case of advanced pregnancy when the theoretical labor zone in the abdominal cavity is quite limited. In fact, in order to minimize the risk of uterine injury, open entry to the peritoneal cavity is generally performed more than 10 cm from the umbilicus, in order to place a trocar (10 or 12 mm) for the optic. Placement of three other conventionally sized trocars (generally 2 x 5 mm trocars and a 10 or 12 mm trocar) in the limited space remaining below the levels of the ribs can then be complicated. In our experience, the use of mini-laparoscopy facilitated trocar placement and did not cause any additional intraoperative difficulties. In addition, postoperative analgesic control was very good, not only thanks to mini-laparoscopy but also thanks to a multimodal approach to pain management. These very preliminary results need to be confirmed in a study, possibly comparative, involving a larger number of patients.

The use of mini-laparoscopy for surgical exploration in pregnant women may be of particular interest at the end of pregnancy, when the patient's abdominal wall could potentially be subjected to severe secondary stress in the event of vaginal delivery. In this limited series of patients who underwent cholecystectomy during late-stage pregnancy, all were able to deliver vaginally at term without difficulty. While it has been fairly well demonstrated that laparoscopic cholecystectomy can be performed in all trimesters of pregnancy under good conditions [6], the risk of inferior obstetric outcomes when surgery is performed in the third trimester has been noted by some authors [8]. For example, Fong et al. reported an increased risk of preterm delivery after cholecystectomy during the third trimester compared with that reported when the decision was made to wait until delivery to schedule this surgery [8]. In the same study, the authors reported a cesarean delivery rate of 44%, but this did not differ from the rate observed when surgery was postponed until after delivery. While the cesarean delivery rate naturally depends on numerous factors linked to the pregnancy, but also to the practices of the centers and even the countries in which the patients are followed, it is interesting to note that all the patients in our study were able to give birth vaginally. The limitation of parietal pain in the weeks following this surgery may have had an impact, albeit limited, on the willingness of obstetric teams and patients to consider natural childbirth.

It is important to point out that any indication for surgery during pregnancy must, of course, be carefully discussed by the obstetrical, surgical, and anesthetic teams. Furthermore, from a general point of view, the selection of patients for MLC is the only guarantee of success. As mini-laparoscopic instruments are thinner and potentially more traumatic, it is difficult to plan to use them in patients with a particularly fragile or thickened gallbladder wall, such as patients with acute or chronic cholecystitis or a history of acute necrotizing pancreatitis. Furthermore, the use of mini-laparoscopy when concomitant surgical extraction of CBD stones is planned appears complex.

## Conclusions

These preliminary results suggest that the mini-laparoscopy could be used safely and without difficulty in selected pregnant women requiring cholecystectomy, even at the end of the second trimester or during the third trimester. The utilization of thin laparoscopic instruments did not result in any significant complications during the operation and may even be of interest in situations where reduced space is required within the abdominal cavity. However, this preliminary study does not allow any conclusions to be drawn, and further larger retrospective and/or prospective comparative studies may be needed to validate the safety of the mini-laparoscopic approach, as well as its efficacy.
